# Comparison of Direct 3D Scans and Plaster Casts in Digital Ankle–Foot Orthosis Workflows

**DOI:** 10.3390/medsci14050589

**Published:** 2026-09-19

**Authors:** Chenxi Peng, Simon Lalor, Nasim Shokoohi, Ling Chen, Erich Rutz

**Affiliations:** 1 CSIRO (Commonwealth Scientific and Industrial Research Organisation) Technology, Clayton South, VIC 3169, Australia; ling.chen@csiro.au; 2 The Royal Children’s Hospital Melbourne, Parkville, VIC 3052, Australia; simon.lalor@rch.org.au (S.L.); nasim.shokoohi@unimelb.edu.au (N.S.); 3 Department of Paediatrics, The University of Melbourne, Parkville, VIC 3052, Australia; 4 Murdoch Children’s Research Institute, Parkville, VIC 3052, Australia; 5 The Hugh Williamson Gait Analysis Laboratory, The Royal Children’s Hospital Melbourne, Parkville, VIC 3052, Australia; 6 Medical Faculty, The University of Basel, 4001 Basel, Switzerland

**Keywords:** ankle–foot orthosis, 3D scanning, plaster cast, additive manufacturing, mesh registration, surface distance

## Abstract

**Background:** Digital ankle–foot orthosis (AFO) workflows depend on accurate lower limb geometry acquisition. Direct 3D scanning on the lower limb may reduce plaster use, but its geometrical agreement with orthotist plaster casting remains insufficiently investigated. **Objective:** This study compared posterior lower limb surfaces captured by direct 3D scanning with surfaces obtained from orthotist plaster casts digitised using the same scanner. **Methods:** Seven healthy paediatric participants underwent bilateral direct scanning and bilateral plaster casting, giving 14 limb comparisons. Cast meshes were digitised, trimmed to reduce boundary effect, globally registered to the direct scans, and segmented into posterior foot, ankle, calf and knee proxy regions. Cast-to-scan distances were then calculated. **Results:** The ankle region showed the greatest residual mismatch, with a weighted mean distance of 3.49 mm (95% CI 3.16–3.81 mm). It also had the highest proportions of vertices exceeding 2 mm (74.8%) and 5 mm (21.2%), and was the highest-error region in 13 of 14 limbs. The knee region had the lowest weighted mean distance (2.28 mm, 95% CI 1.92–2.60 mm) but showed the largest upper-tail spread. **Conclusions:** These preliminary findings indicate direct scanning and plaster casting did not produce completely interchangeable posterior limb surfaces in this cohort. The ankle was the most consistently discrepant AFO-relevant region, indicating that digital AFO workflows should include local geometric quality control rather than relying only on whole-limb agreement.

## 1. Introduction

**Figure 1:**
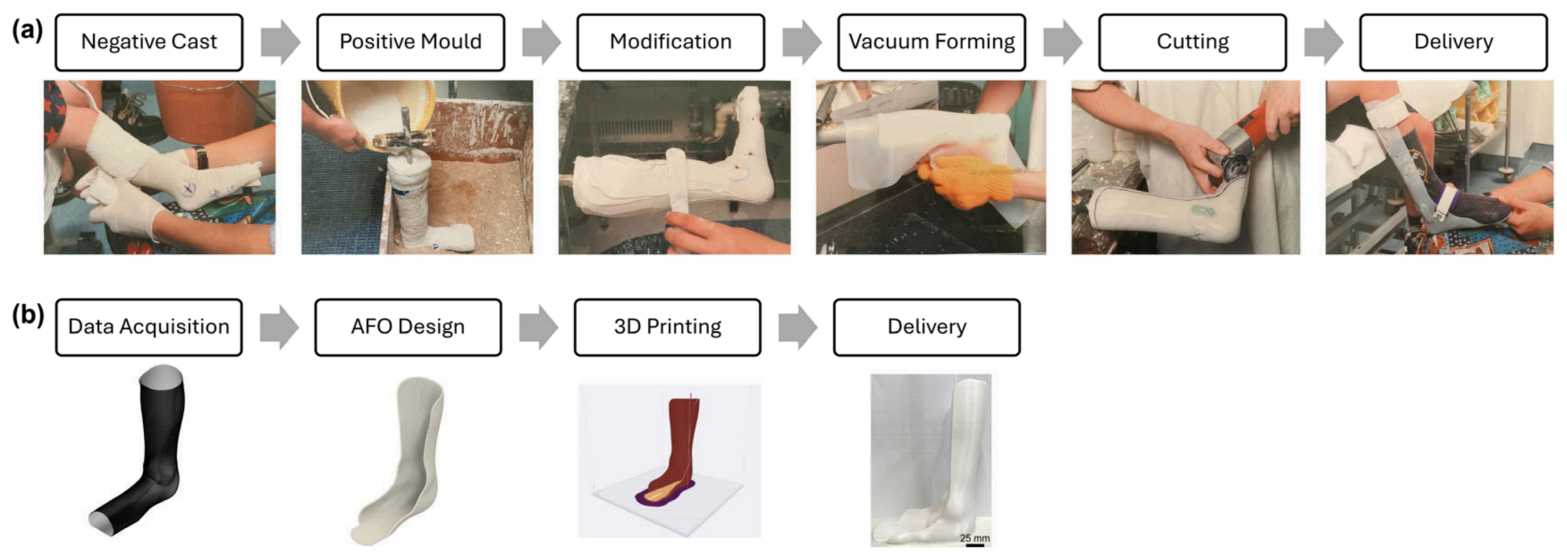
Traditional and digital AFO workflows. (**a**) Traditional fabrication begins with plaster or fibreglass casting, positive-model preparation and thermoforming. (**b**) Digital fabrication uses 3D scanning, computer-aided design and additive manufacturing to produce the orthosis [29].

Custom ankle–foot orthoses (AFOs) are prescribed to support the foot, ankle and lower leg in children with neuromuscular or musculoskeletal impairments [1,2,3,4]. Common clinical goals include improving toe clearance, maintaining foot and ankle alignment, accommodating deformity, supporting stance phase stability and controlling pathological ankle motion [5,6,7]. Conventional custom fabrication, as presented in Figure 1a, usually begins with a plaster or fibreglass negative impression, followed by creation and manual modification of a positive model before thermoforming, trimming and fitting [8,9]. This workflow remains clinically established, but it is labour-intensive, material-consuming, difficult to reproduce consistently and heavily dependent on orthotist judgement during both casting and model rectification [10,11,12].

Digital AFO workflows have been proposed to mitigate those issues [13,14,15]. These approaches generally acquire patient-specific geometry through 3D scanning or related imaging, reconstruct a surface model, design the orthosis in computer-aided design (CAD) software and manufacture the device using additive manufacturing (Figure 1b) [16,17]. Reviews of 3D-printed AFOs suggest potential advantages in customisation, weight reduction, stiffness tuning, and production efficiency [18]. However, clinical evidence remains heterogeneous, often with small samples, short follow-up and variable outcome measures [19]. Early and contemporary studies have also shown that printed or digitally designed AFOs can be produced and evaluated, but this does not eliminate the need to validate the input geometry [13,18]. The quality of the captured surface is therefore a central issue. Scanner technology, operator technique, scanning time, subject motion, limb posture, software processing and the clinical environment can all affect the resulting mesh [20,21,22]. Similar concerns have been raised in foot-scanning studies, where protocol variability and incomplete reporting limit comparison across studies [23,24]. These issues are particularly relevant for AFOs because the required geometry extends beyond the foot to the ankle and lower leg, and device performance is strongly affected by local design features [25,26,27,28].

Previous studies comparing 3D scanning and traditional morphology capture are encouraging but incomplete. A systematic review found that scanning can be faster than casting, particularly for experienced users, but that accuracy and reliability vary across studies and anatomical measures [3]. More AFO-relevant studies have reported promising reliability and validity for limb scanning and promising speed and accuracy when scanners were compared with plaster casting in children requiring AFOs [20,24]. Most evidence, however, remains based on global dimensions, landmark type measures or workflow feasibility. Less is known about regional surface agreement across relevant limb areas. This distinction matters because plaster casting is not a passive measurement procedure. During casting, the orthotist may reposition the limb, compress soft tissue, smooth the surface and shape the geometry. Traditional cast modification can also be spatially structured and patient-specific. Consequently, differences between a direct scan and a plaster cast may reflect scanning error, posture, soft-tissue deformation, orthotist handling, cast processing or genuine differences between the two workflows. To our knowledge, no prior study has quantified regional scan-to-cast agreement while separately accounting for residual whole-limb registration error. Without this step, it is unclear whether reported regional differences reflect genuine shape mismatch or simply leftover misalignment. This study addresses that gap: distance is calculated before and after each anatomical region is realigned, and the results distinguish regions with broad surface mismatch from those where disagreement is confined to a smaller, localised area.

This study compared lower limb geometry of children obtained by direct 3D scanning with geometry obtained from digitising orthotist plaster casts. We focused on posterior limb regions that are relevant to orthosis fit and function, including the foot, ankle, calf and knee. The scan-to-cast agreement was quantified and analysed in different anatomical regions. The findings could provide insight into the development and implementation of future digital and 3D-printed AFO workflows.

## 2. Materials and Methods

### 2.1. Study Design and Data Acquisition

Seven healthy, typically developing paediatric participants were recruited for a bilateral lower limb comparison. Ethical approval for this study was obtained from the University of Melbourne Human Research Ethics Committee (approval number: 2024-29818-57655-3, approval date: 30th August 2024). Written consent was obtained from participants and their parents or guardians. Both legs of each participant were included, giving 14 limb comparisons. Participant demographic characteristics are summarised in Table 1.

Each lower limb was captured using the 3dMDflex system (3dMD, Atlanta, GA, USA). The 3dMDflex is a multi-camera stereophotogrammetry system that captures a synchronised set of surface images and reconstructs them as a 3D skin surface mesh. The reconstructed surface was exported as a standard tessellation language (STL) file for subsequent processing. During direct 3D scanning, participants were positioned supine on a plinth with the hip and knee flexed to 90 degrees. Participants were instructed to maintain a plantargrade ankle and forefoot position with the rearfoot in neutral (target 0° inversion/eversion) during capture.

**Figure 2:**
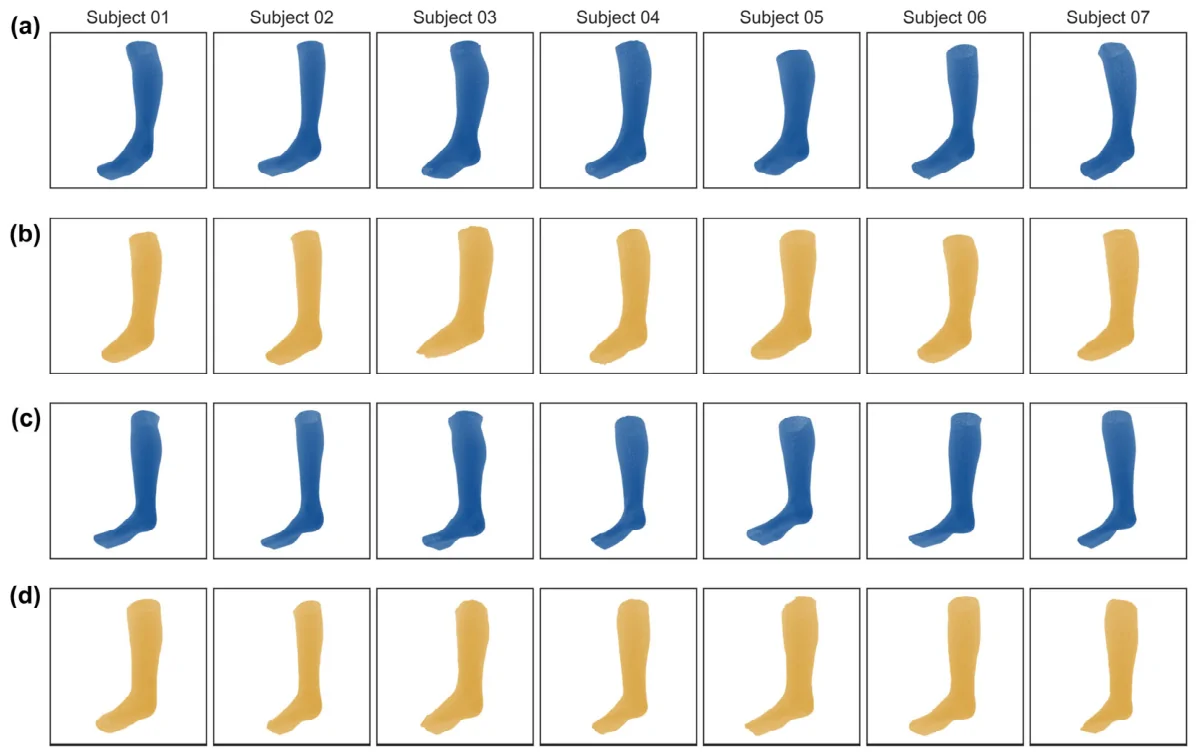
De-identified direct scan and digitised plaster cast meshes for all seven participants. Rows (**a**,**c**) show direct scans (blue), while rows (**b**,**d**) show the corresponding digitised casts (yellow) for the two limbs of each participant.

A single senior orthotist then produced a plaster or fibreglass cast of the same limb. The cast of each limb was taken with the participant sitting in a chair with the hips and knees flexed to 90 degrees. A semi-weight-bearing cast was taken using a flat footboard, with the ankle maintained at plantargrade and the rearfoot and forefoot maintained in neutral alignment. No corrective loading or pressure was deliberately applied over the malleoli or ankle region. Each cast was subsequently digitised using the same 3D scanning system. The direct scan and digitised cast were subsequently overlaid in Canfit (Vorum Research Corporation, Vancouver, BC, Canada) for visual assessment by the orthotist. Adjustments were limited to translation and rotation of each mesh as a whole, while joint positions and surface shapes were not digitally modified. The different postures used for direct scanning and casting meant that gravitational loading and compressive loading on the soft tissues were not identical between conditions. Direct scanning was performed supine and unloaded, while casting was performed seated and semi-weight-bearing. Any distance between the two surfaces therefore reflects posture and loading, not acquisition method alone. The direct scan and digitised plaster cast meshes for all seven participants are presented in Figure 2. 

### 2.2. Mesh Preprocessing and Global Registration

The direct scan and digitised cast for each limb were exported as binary STL surface meshes. The direct scan was treated as the fixed target surface and the digitised cast as the moving source surface. All coordinates and distances were analysed in millimetres. Registration used rigid transformations only, so a cast point was transformed as

(1) $$\;x^{\prime }=xR^{T}+t,\;$$

where ***R*** is a 3 × 3 rotation matrix, and ***t*** is a translation vector. No scaling or non-rigid deformation was applied.

The initial whole-limb registration used area-weighted random surface samples from both meshes. For each mesh, 12,000 points were sampled from triangular faces with selection probability proportional to face area. A coarse alignment was obtained by matching the principal component frames of the cast and scan samples. Candidate axis permutations and sign combinations were evaluated to address ambiguity in principal components. The candidate with the lowest mean nearest-neighbour distance was selected for initialisation. The cast sample was then refined to the scan sample using rigid iterative closest point (ICP) registration for a maximum of 35 iterations. At each iteration, the nearest scan neighbours were assigned to transformed cast points. The optimal rigid update was computed using the Kabsch singular value decomposition solution. Iteration stopped early if the change in mean nearest-neighbour distance was less than 10^−7^ mm. The final transformation was applied to all cast STL vertices.

After this initial registration, boundary regions likely to represent scan edge artefacts were removed. The same rule was applied in scan coordinates to both meshes. Vertices above the positive z trim and vertices close to the negative y boundary were excluded. The final correction used a positive z trim of 15 mm and a negative y trim of 5 mm. The trimmed cast surface was then re-registered to the trimmed scan surface using rigid ICP initialised with identity rotation and zero translation. This second registration reduced the influence of scan boundaries on the final alignment.

### 2.3. Posterior Anatomical Regions

The analysis focused on posterior limb geometry because this surface is central to many AFO shell and fit characteristics. Four anatomical regions were defined on the trimmed meshes: foot, ankle, calf and knee. The z coordinate of the trimmed scan was normalised from 0 to 1 using the minimum and maximum z coordinates of the trimmed scans. Regions were assigned using proportional longitudinal bands: foot, 0.00–0.12; ankle, 0.12–0.32; calf, 0.32–0.68; and knee, 0.68–1.00 of the normalised z range. Posterior selection was defined using scan-derived y thresholds within each longitudinal band. For the foot and ankle, the 70th percentile of scan vertex y coordinates was used, retaining the most posterior 30% of vertices. For the calf and knee, the median y coordinate was used, retaining the posterior half of each band. The region definition was assessed qualitatively through sensitivity analysis on all 14 direct-scan meshes and are presented in Supplementary Section S1.

The same scan-derived thresholds were applied to the aligned cast mesh, so scan and cast regions were defined in a common coordinate system. Region definitions for all subjects were visually checked by a consultant paediatric orthopaedic surgeon. This approach provided a reproducible coordinate-based method for comparing the same relative limb regions. However, proportional segmentation may map differently onto absolute anatomy across a wide paediatric age range because limb length, foot size and soft-tissue morphology change with growth. The segmented regions should therefore be interpreted as anatomical proxy regions rather than exact landmark-defined regions.

### 2.4. Local Regional Realignment

Regional distances were not calculated directly after whole-limb registration. Each segmented region was locally realigned before distance calculation. For each region, posterior scan vertices and posterior cast vertices were extracted from the trimmed, globally aligned meshes. If both surfaces contained at least 20 vertices, the cast region was translated so that its centroid matched the scan-region centroid. It was then refined using rigid ICP for a maximum of 25 iterations. This local realignment was included to reduce confounding between residual whole-limb registration error and local shape difference. The regional metrics report residual mismatch after each region was allowed an independent rigid alignment. This choice was made because preliminary analyses showed that regional comparisons based only on whole-limb alignment could overstate differences caused by residual registration error. Distances were also obtained from the final whole-limb-registered geometry without additional regional translation or ICP and are presented in Supplementary Section S2.

### 2.5. Distance Metrics and Statistical Summaries

Distances were computed as nearest-neighbour vertex distances. For the primary regional analysis, locally aligned cast vertices were used as query points and direct-scan vertices were used as the reference surface. For a cast region vertex and the set of scan-region vertices, distance was defined as

(2) $$d_{i}=\underset{s_{j}\in S}{\min}{\left\|c_{i}-s_{j}\right\|}_{2}.\;$$

Nearest-neighbour queries used a k-d tree. Distances were unsigned, so they quantified mismatch magnitude but not whether the cast surface lay inside or outside the direct scan. Cast-to-scan distances were used for the main regional results and distance maps. Scan-to-cast distances were also calculated during quality checking to assess one-sided nearest-neighbour effects. For each limb and region, cast-to-scan distance distributions were summarised using central tendency, spread, upper-tail and threshold metrics. Extracted values included vertex count, mean, median, standard deviation, root mean square (RMS) distance, 95th percentile (P95), 99th percentile (P99), maximum distance, and proportions of vertices within 1 mm, 2 mm, 3 mm and 5 mm. RMS distance was calculated as

(3) $$RMS=\sqrt{\frac{1}{n}\sum_{i=1}^{n}d_{i}^{2}}.\;$$

Threshold exceedance was reported as the complementary proportion above each threshold. For example, 5 mm exceedance was calculated as 100 × (1 − $p_{\leq 5\;mm}$), where $p_{\leq 5\;mm}$ is the proportion of vertices with $d_{i}\leq 5.00\;mm$.

Cohort-level regional means were weighted by the number of cast vertices in each region. Bootstrap confidence intervals were calculated across limb-level regional values. Left–right side effects were calculated within participants for each region and summarised using bootstrap confidence intervals and Wilcoxon signed-rank tests. Subject heterogeneity was quantified as the range of mean distances across leg–region combinations. Tail shape was described using P95 minus median distance and the P95 to median ratio.

### 2.6. Directional Difference Analysis

Directional differences were evaluated using the nearest points on a direct-scan triangle-surface patch. For each cast vertex, signed normal displacement was calculated by projecting the vector from the nearest surface point to the vertex onto the scan normal. Normals were recomputed, consistently oriented outward and interpolated at the nearest surface point. Positive and negative values denoted outward and inward cast displacement relative to the scan, respectively. Values within ±0.05 mm were classified as near zero. Predominant outward or inward displacement was defined as at least 60% of vertices above +0.05 mm or below −0.05 mm, respectively. Otherwise, the limb was classified as mixed. Cohort summaries were vertex-weighted, and confidence intervals were estimated using the limb-level bootstrap described above. Normal-orientation checks and additional implementation details are provided in Supplementary Section S3.

## 3. Results

### 3.1. Regional Geometric Agreement

**Figure 3:**
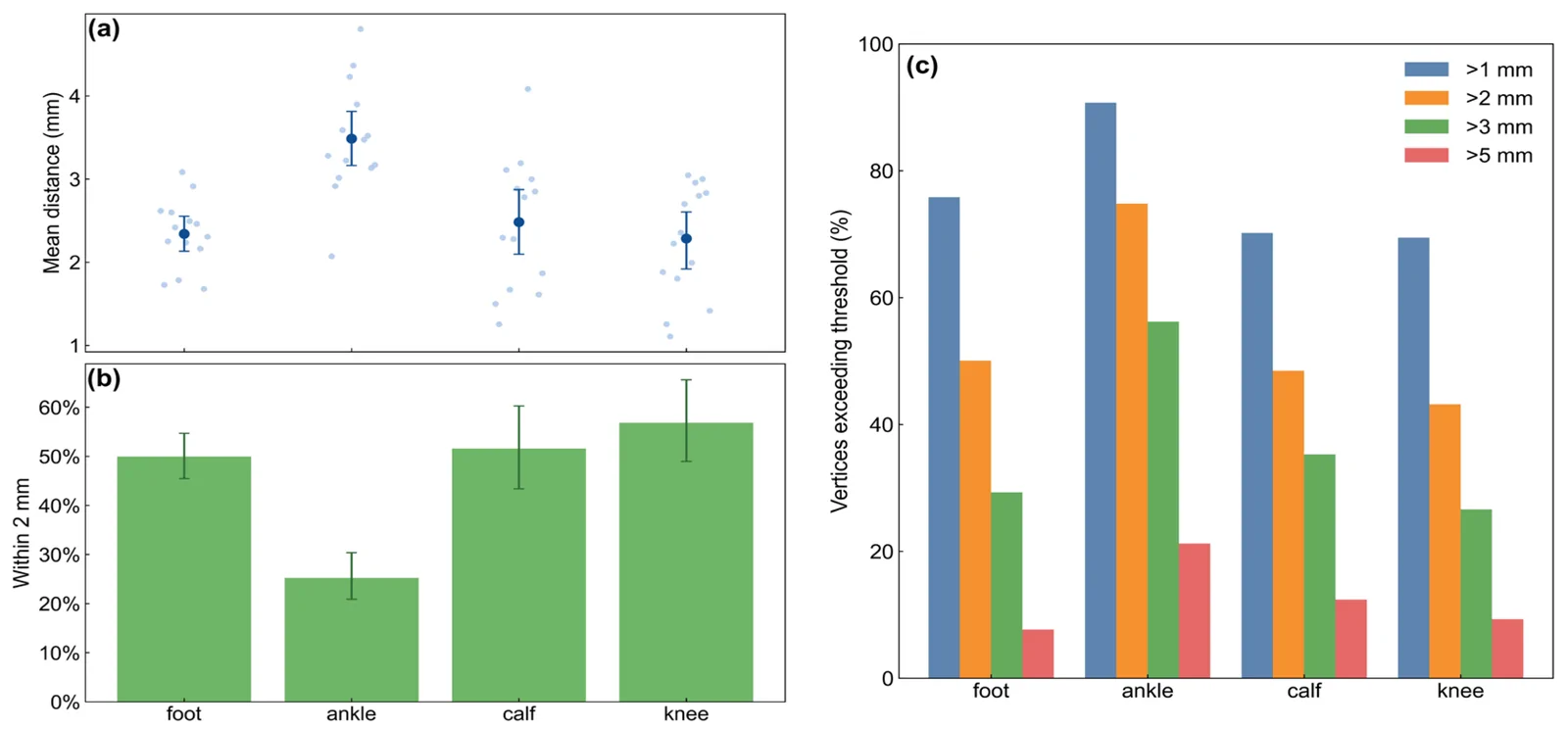
(**a**) Limb-level mean distances with weighted regional means and 95% confidence intervals; (**b**) percentage of vertices within 2 mm; and (**c**) percentage of vertices exceeding 1, 2, 3 and 5 mm thresholds.

First, a surface agreement analysis was performed between direct limb scans and corresponding plaster cast scans across fourteen limb pairs to quantify regional geometric differences between the two acquisition methods. Table 2. provides a regional distance summary based on 14 limb comparisons. As presented in Figure 3a, the regional agreement varied across the posterior limb surface. The ankle had the largest residual mismatch, with a weighted mean distance of 3.49 mm (95% CI 3.16–3.81 mm). It also had the largest P95 distance (6.83 mm). The knee had the smallest weighted mean distance (2.28 mm, 95% CI 1.92–2.60 mm) but the largest P99 distance (9.10 mm). The foot and calf showed intermediate weighted mean distances of 2.34 mm and 2.48 mm, respectively. Regional agreement varied substantially, ranging from relatively close correspondence at the knee (56.8% of vertices within 2 mm) to consistently large mismatch at the ankle (25.2% within 2 mm).

### 3.2. Threshold Exceedance

**Figure 4:**
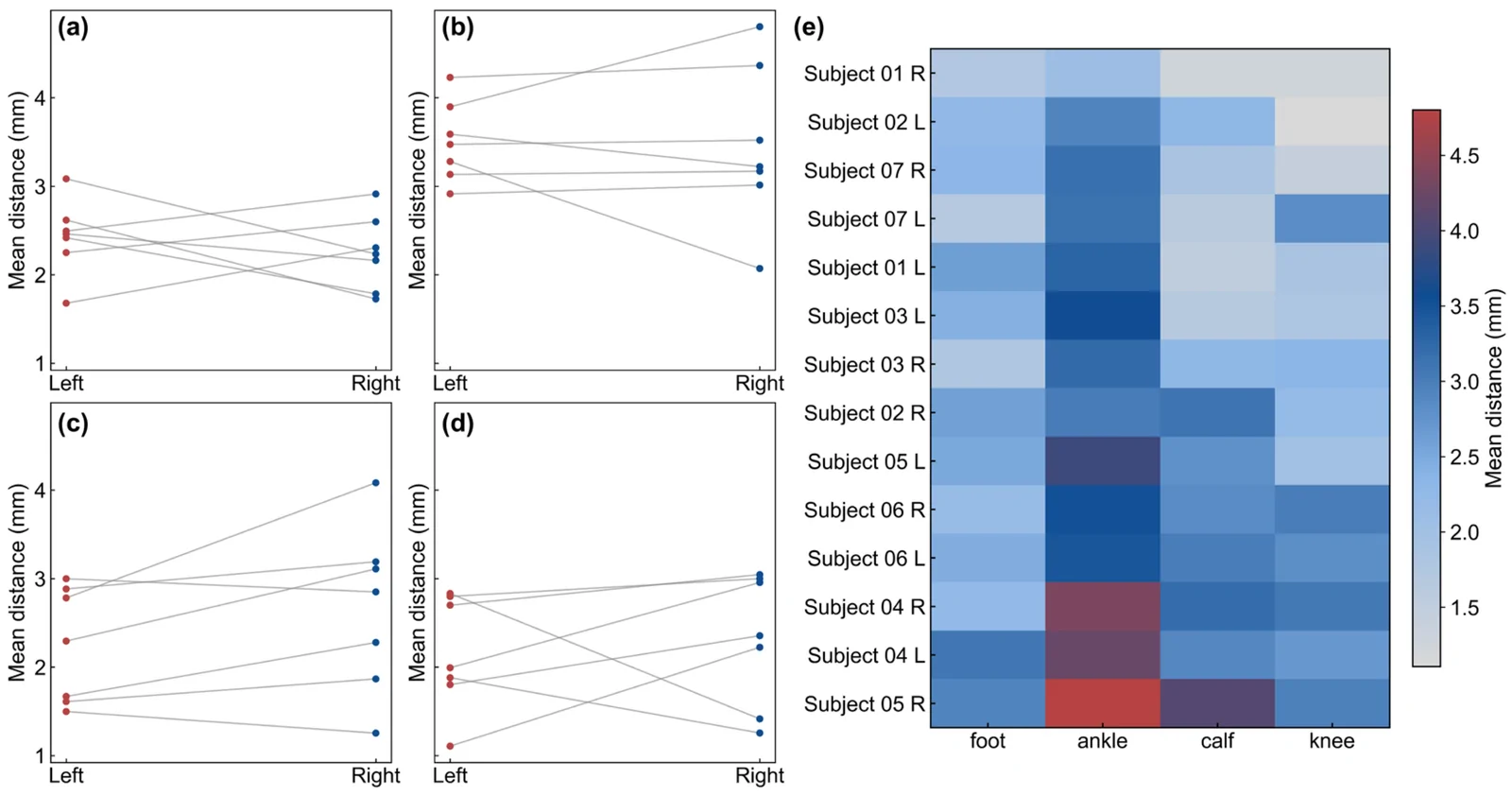
Limb-level and regional consistency. (**a**–**d**) Left–right mean distances for the foot, ankle, calf and knee regions, respectively. (**e**) Heat map of mean distance by limb and anatomical region.

The threshold analysis of surface agreement between direct scans and plaster casts was performed with the aim of quantifying the proportion of surface vertices falling within predefined analytical distance thresholds. Figure 3b,c show results of the threshold analysis. The ankle had the lowest proportion of vertices within 2 mm (25.2%) and the highest proportions exceeding 2 mm (74.8%) and 5 mm (21.2%). The knee had the highest proportion within 2 mm (56.8%) and a lower proportion exceeding 5 mm (9.3%). The foot and calf had 50.1% and 48.5% of vertices exceeding 2 mm, respectively. Overall, the threshold analysis confirmed regional differences in surface agreement, with the ankle displaying the greatest geometric deviation and the knee the closest correspondence between scanning methods. These findings are consistent with the distance-based analysis shown in Figure 4a, indicating that discrepancies were concentrated primarily around the ankle region.

### 3.3. Consistency of the Regional Pattern

To determine whether scanning discrepancies were influenced by limb side and to evaluate inter-subject variability, the consistency of regional surface agreement between left and right limbs was examined. Left–right comparisons showed no strong systematic side difference in most regions (Figure 4a–d). The mean left–right difference was 0.18 mm for the foot, 0.05 mm for the ankle and −0.16 mm for the knee. The largest side effect was observed in the calf, where the mean left–right difference was −0.41 mm (bootstrap 95% CI −0.80–0.06 mm; Wilcoxon *p* = 0.078). The ankle was the highest-error region in 13 of 14 limb comparisons. This pattern was also largely consistent within participants. For six of seven participants, the ankle was the worst region in both the left and right legs. In the remaining participant, one leg had the calf as the highest-error region. The heat map of limb-level regional mean distances showed subject-to-subject variation in absolute mismatch, but the dominant ankle pattern remained visible across the cohort (Figure 4e). Subject-level mean regional distance ranged from 1.95 mm to 3.24 mm. The highest mismatch subject also showed the greatest regional heterogeneity, with a 2.81 mm range across leg–region combinations. Overall, the results indicate that measurement differences were largely independent of limb side, while consistently identifying the ankle as the region most susceptible to mismatch. Although the magnitude of mismatch varied between participants, the regional pattern remained remarkably consistent across the cohort.

### 3.4. Sensitivity to Region Definition

Across the 27 ankle-proxy definitions, the weighted mean distance after local realignment ranged from 3.40 to 3.71 mm, compared with 3.49 mm at baseline. The maximum absolute deviation was 0.23 mm (6.55%), while one-at-a-time changes were at most 0.11 mm (3.22%). The weighted mean limb-level P95 ranged from 6.59 to 7.55 mm. Full results are provided in Supplementary Section S1.

### 3.5. Sensitivity to Regional Alignment

After whole-limb registration alone, weighted mean distances were 3.97 mm for the foot, 5.64 mm for the ankle, 4.59 mm for the calf and 3.36 mm for the knee. The ankle had the highest mean distance in 11 of 14 limbs. Local realignment reduced the mean distance in all 56 limb–region comparisons. Mean paired reductions were 1.62, 2.20, 2.10 and 1.06 mm for the foot, ankle, calf and knee, respectively. Full results are provided in Supplementary Section S2.

### 3.6. Directional Difference Analysis Results

The weighted mean signed normal displacement was +2.70 mm (95% CI +2.44 to +2.92 mm). Across 143,673 cast vertices, 86.3% were displaced outward, 13.2% inward and 0.5% were within ±0.05 mm of zero. All 14 limbs met the criterion for predominantly outward displacement, with limb-level means ranging from +1.41 to +3.38 mm (Supplementary Section S3).

## 4. Discussion

This study demonstrated that direct lower limb 3D scanning and orthotist plaster casting can produce measurably different posterior limb surfaces. These differences remained evident even though the casts were digitised using the same scanner and each anatomical region was independently realigned before distance calculation. The principal finding was strongly regional: the ankle had the largest residual mean distance, the highest threshold exceedance and the highest-error ranking in 13 of the 14 limbs. This consistent pattern identifies the ankle as an important region for geometric quality control in digital AFO workflows. The magnitude and distribution of the ankle disagreement suggest that it was not attributable solely to small, randomly distributed surface noise. The ankle had a weighted mean distance of 3.49 mm, and 21.2% of its vertices exceeded 5 mm. The 3.49 mm ankle distance cannot be attributed purely to scanning versus casting method, since posture and loading differed between the two acquisition conditions. Some portion of it plausibly reflects the unloaded, supine scan being compared against a semi-weight-bearing, seated cast. Meanwhile, because the clinical significance of the selected distance thresholds has not yet been established, these values should be interpreted as comparative reference points rather than confirmed clinical tolerances. Nevertheless, the consistently greater threshold exceedance at the ankle indicates that the disagreement was spatially concentrated and region-dependent.

Directional analysis showed that the ankle difference was predominantly outward relative to the direct-scan surface. This tendency was observed in all 14 limbs, with a weighted mean normal displacement of +2.70 mm and 86.3% of vertices displaced outward. The remaining inward displacements indicate local variation within this overall pattern. Because the analysis followed independent rigid ankle realignment, it describes residual surface geometry rather than the position of the ankle relative to the rest of the limb. The signed normal projection also complements, rather than directly decomposes, the primary unsigned nearest-neighbour distance.

The upper-tail analysis provided additional information about the form of the disagreement. The ankle demonstrated the largest average mismatch and the greatest threshold exceedance, indicating relatively broad regional disagreement. In contrast, the knee had a lower mean distance but more pronounced upper-tail behaviour relative to its median, suggesting that its errors were more localised. This distinction is important for workflow development. Broad regional mismatch may require improvements in acquisition protocol, limb positioning or region-specific design allowances, whereas localised upper-tail errors may be better addressed through targeted mesh-completeness checks near anatomical features or scan boundaries. The regional pattern was also broadly consistent across limbs and participants. Mean regional distance varied between participants from 1.95 to 3.24 mm, indicating some subject-specific heterogeneity. This variation may reflect differences in anatomy, limb morphology, soft-tissue characteristics, scan quality or casting technique. However, the ankle remained the highest-error region in nearly all limbs, and the highest-error region was the same on both sides in 85.7% of participants. These findings suggest that the dominant ankle pattern was not caused by a single participant or an isolated poor-quality scan.

Several mechanisms may explain why the ankle was particularly susceptible to disagreement. The biomechanical properties of the tissues overlying the ankle may have contributed to the observed surface differences. The medial and lateral malleoli are covered by relatively thin layers of skin and adipose tissue. Adipose tissue exhibits viscoelastic behaviour under loading, meaning that sustained compression can produce time-dependent deformation [30]. During plaster or fibreglass casting, an orthotist applies compressive and corrective forces over several minutes while capturing the shape of the limb. This process may induce tissue creep and displace the external surface from its unloaded resting geometry. Consequently, the resulting cast may represent a loaded and time-averaged tissue configuration, whereas the direct 3D scan captures a more instantaneous and less compressed surface. The tissue-viscoelasticity explanation above accounts for part of the ankle finding, but not necessarily all of it. The observed ankle mismatch could reflect unwanted geometric disagreement with tissue creep under sustained contact, incomplete scan capture around the malleoli, or the posture and loading confound described in the Limitations. It could also reflect intentional clinical shaping, since the rearfoot was physically held in neutral throughout casting. Here, the casting protocol gives a clear answer for at least one part of this question, with no corrective loading or pressure being applied over the malleoli or ankle region specifically. This rules out active correction as an explanation for the ankle mismatch itself, even though neutral positioning was maintained elsewhere in the limb. The ankle finding is therefore more plausibly explained by tissue creep, scan completeness or the posture and loading confound rather than by the intended correction at that site.

The anatomical complexity of the ankle may further amplify these effects. The region contains prominent malleoli, narrow contours and recessed or concave surfaces. These features can be difficult to capture completely using optical scanning and may also be susceptible to infilling or bridging by plaster or fibreglass bandage during casting and manipulation. Similar challenges in capturing complex foot and ankle geometry have been noted in the surface-scanning literature [21,22]. In contrast, the calf contains a greater volume of muscle and soft tissue, which may distribute applied forces more broadly and reduce localised surface displacement. The persistent ankle mismatch observed in this study is therefore consistent with known regional differences in anatomy, tissue thickness and loading response.

Orthotist casting technique is another plausible source of disagreement. Casting was performed by a single senior orthotist, which likely improved procedural consistency within the study but did not permit assessment of inter-operator variability. Differences between orthotists in limb handling, corrective force application, bandage tension, contouring and cast removal could alter the resulting surface geometry. Previous studies of orthotic morphology capture have reported variability between clinicians and between repeated casting procedures, although direct evidence for paediatric AFO casting remains limited [31]. The use of one orthotist should therefore be regarded as both a strength for internal consistency and a limitation for generalisability.

Differences in participant posture and limb loading between the two acquisition procedures may also have contributed to the mismatch. Direct scanning was performed with the participant supine, whereas plaster casting was performed with the participant sitting in a semi-weight-bearing configuration. Although both protocols aimed to maintain a plantargrade ankle and neutral rearfoot and forefoot alignment, the gravitational forces, muscle activity and contact pressure against the footboard were not equivalent. The comparison, therefore, reflects not only differences between scanning and casting methods, but also differences in the mechanical state of the limb during acquisition. Future studies should standardise posture and loading more closely or deliberately compare matched and unmatched positioning protocols.

Taken together, these findings extend the digital AFO literature from assessments of feasibility and global accuracy towards region-specific surface agreement. Previous studies indicate that 3D scanning and additive manufacturing may improve aspects of AFO production, while also reporting variability in accuracy, reliability and clinical evidence [12,20]. The present results show that whole-limb agreement alone may obscure important local differences. A surface may show acceptable global registration while retaining substantial disagreement around the ankle or other clinically important regions. The findings should not be interpreted as evidence that direct scanning is unsuitable for digital AFO fabrication, nor should plaster casting necessarily be treated as an error-free geometric reference. Rather, the two methods capture the limb under different physical and procedural conditions and should not be assumed to produce interchangeable surfaces. These findings raise the hypothesis that digital AFO workflows could benefit from region-specific quality-control procedures, particularly around the malleoli and posterior ankle, although this remains to be tested against clinical fit and functional outcomes. Practical measures could include standardised limb positioning, planned scanner trajectories around recessed anatomy, immediate checks for incomplete surface capture and region-specific design allowances. Future work should combine repeated casting by multiple orthotists, matched limb-position protocols, real-time pressure measurement and finite element modelling of soft-tissue deformation. These approaches would help distinguish the relative contributions of tissue viscoelasticity, posture, scan incompleteness and operator-dependent shaping to the geometric disagreement observed in this study.

The geometric agreement reported here is only the first step in a much longer chain. How closely the direct scan and the casts agree determines the shape of the manufactured AFO, and that in turn determines the fit and pressure distribution. Fit and pressure distribution determine the anatomical ankle alignment during standing and gait, and alignment during gait determines the wearer’s function, comfort and satisfaction. This study addresses only that first step.

## 5. Limitations

Several limitations constrain interpretation of this work. First, the sample was small, with seven participants and 14 limbs. The results should therefore be viewed as foundational rather than definitive. The width of the bootstrap confidence intervals is a direct consequence of having only 14 limbs, so those intervals are expected to narrow as future studies increase the number. Second, posture and loading were not matched between the two acquisition methods, with direct scanning positioned supine and unloaded while casting was seated and semi-weight-bearing. This is a primary limitation rather than a secondary one, since it confounds any attempt to attribute regional mismatch, particularly at the ankle, to acquisition method alone. Positioning verification relied on the orthotist’s qualitative visual assessment of overlaid meshes in Canfit and did not provide objective, quantitative confirmation of ankle angle. Although whole-object translation and rotation facilitated surface comparison, these adjustments could not establish equivalent joint positioning during acquisition. Residual differences in ankle position, together with differences in posture and loading, may therefore have contributed to the observed mismatch, particularly at the ankle. Third, the regions were geometric proxy regions rather than landmark-defined anatomical regions. This improved reproducibility but may not perfectly match clinical landmarks such as the malleoli or calcaneal borders. Fourth, results remain dependent on scan completeness, mesh quality, trimming choices and segmentation thresholds, despite the use of visual checks and sensitivity analysis. Fifth, participants in this study were typically developing children, without diagnosed neuromuscular or musculoskeletal impairment, and not the population AFOs are actually prescribed for. This matters most for the tissue viscoelasticity and casting technique discussion in this work, since soft-tissue behaviour and casting difficulty in spasticity, contracture or fixed deformity may differ substantially from a typically developing limb. This study also collected no biomechanical or functional data, so the geometric mismatch reported cannot yet be linked to any real difference in fit, pressure distribution or gait outcomes.

## 6. Conclusions

This study aimed to quantify regional geometric agreement between direct lower limb 3D scanning and orthotist plaster casting, in seven participants across 14 limbs. These preliminary findings indicate that direct scanning and plaster casting did not produce interchangeable posterior limb surfaces in this cohort. The discrepancy was strongly region-dependent. The ankle showed the largest mismatch, with a weighted mean distance of 3.49 mm (95% CI 3.16–3.81 mm) and 74.8% of its vertices exceeding 2 mm. The knee fared better, with 56.8% of vertices falling within 2 mm. The ankle was the highest-error region in 13 of the 14 limbs, not an isolated result from one outlying subject. Digital and 3D-printed AFO workflows should therefore include local surface agreement checks, not just whole-limb registration error. Fit, suspension, trimline placement and pressure distribution are plausibly relevant to local geometry, although this was not evaluated in the present study. Local analysis also gives a more defensible way to separate genuine shape mismatch from residual whole-limb alignment error. Direct 3D scanning remains a promising route toward cleaner, more reproducible AFO production. But replacing plaster casting with it should be supported by region-specific validation, not assumed. Future studies should test larger, clinically diverse cohorts, examine soft-tissue deformation during casting, and determine how regional geometric mismatch affects AFO fit, comfort, adjustment burden and functional outcomes.

## Figures and Tables

**Table 1:** Demographic characteristics of participants in the study.

Characteristic	n	Mean	Median	Range
Age (years)	7	10.7	11.0	6.7–15.0
Height (cm)	7	143.1	152.0	118–158
Weight (kg)	7	38.3	38.0	22–54

**Table 2:** Regional distance summary based on 14 limb comparisons.

Region	Mean (mm)	95% CI Low (mm)	95% CI High (mm)	P95 (mm)	P99 (mm)	Within 2 mm (%)
foot	2.34	2.13	2.55	5.31	6.55	49.9
ankle	3.49	3.16	3.81	6.83	8.35	25.2
calf	2.48	2.10	2.87	5.50	7.01	51.5
knee	2.28	1.92	2.60	5.13	9.10	56.8

## Data Availability

The data presented in this study are available on request from the corresponding author. The data are not publicly available due to ethical restrictions.

## Supplementary Materials

The following supporting information can be downloaded at: https://www.mdpi.com/article/10.3390/medsci14050589/s1, Figure S1: Panels a-n show mediolateral-height projections of the lower 45% of the trimmed direct-scan mesh for the left and right limbs of seven participants. Grey points show the complete trimmed mesh, red points show the posterior ankle proxy, and dashed lines show its inferior and superior proportional boundaries (0.12 and 0.32 of normalised scan height).; Figure S2: Sensitivity of ankle-region results to the anatomical proxy definition. All 27 combinations of lower normalised-height boundary (0.10, 0.12, or 0.14), upper boundary (0.30, 0.32, or 0.34), and posterior surface retained (25%, 30%, or 35%) were evaluated across 14 limbs. The baseline was 0.12–0.32 with 30% posterior retention. (a) Change in cast-vertex-weighted cohort mean distance relative to baseline. (b) Mean limb-level cast–-mask Jaccard overlap with baseline. (c) Limb-level mean-distance changes for one-at-a-time variations; horizontal bars show limb means.; Table S1: Regional mean distance under whole-limb registration and local realignment (uUnit in mm).; Table S2: Signed normal displacement across limbs.

## Abbreviations

The following abbreviations are used in this manuscript:

AFO	Ankle–foot orthosis
CI	Confidence interval
CAD	Computer-aided design
3D	Three dimensional
STL	Standard Tessellation Language
ICP	Iterative closest point
RMS	Root mean square
P95	95th percentile
P99	99th percentile

## References

[1] Graham H.K., Rosenbaum P., Paneth N., Dan B., Lin J.P., Damiano D.L., Becher J.G., Gaebler-Spira D., Colver A., Reddihough D.S. (2016). Cerebral palsy. Nat. Rev. Dis. Primers.

[2] Lintanf M., Bourseul J.S., Houx L., Lempereur M., Brochard S., Pons C. (2018). Effect of ankle-foot orthoses on gait, balance and gross motor function in children with cerebral palsy: A systematic review and meta-analysis. Clin. Rehabil..

[3] Graham H.K., Thomason P., Willoughby K., Hastings-Ison T., Stralen R.V., Dala-Ali B., Wong P., Rutz E. (2021). Musculoskeletal Pathology in Cerebral Palsy: A Classification System and Reliability Study. Children.

[4] Korbal T., Graham K., Thuraisingam S., Howard J.J., Rutz E. (2025). Epidemiology of Lower Limb Musculoskeletal Pathology in Cerebral Palsy: A Population-Based, Cohort Study. JB JS Open Access.

[5] Aboutorabi A., Arazpour M., Ahmadi Bani M., Saeedi H., Head J.S. (2017). Efficacy of ankle foot orthoses types on walking in children with cerebral palsy: A systematic review. Ann. Phys. Rehabil. Med..

[6] Ridgewell E., Dobson F., Bach T., Baker R. (2010). A systematic review to determine best practice reporting guidelines for AFO interventions in studies involving children with cerebral palsy. Prosthet. Orthot. Int..

[7] Eddison N., Mulholland M., Chockalingam N. (2017). Do research papers provide enough information on design and material used in ankle foot orthoses for children with cerebral palsy? A systematic review. J. Child. Orthop..

[8] Zhou C., Yang Z., Li K., Ye X. (2022). Research and Development of Ankle-Foot Orthoses: A Review. Sensors.

[9] Schröder J.-H., Barandun G.A., Leimer P., Morand R., Göpfert B., Rutz E. (2024). Novel Modular Walking Orthosis (MOWA) for Powerful Correction of Gait Deviations in Subjects with a Neurological Disease. Children.

[10] Wang J.Z., Lillia J., Farhan M., Bi L., Kim J., Burns J., Cheng T.L. (2021). Digital mapping of a manual fabrication method for paediatric ankle-foot orthoses. Sci. Rep..

[11] Wojciechowski E., Chang A.Y., Balassone D., Ford J., Cheng T.L., Little D., Menezes M.P., Hogan S., Burns J. (2019). Feasibility of designing, manufacturing and delivering 3D printed ankle-foot orthoses: A systematic review. J. Foot Ankle Res..

[12] Farhan M., Wang J.Z., Bray P., Burns J., Cheng T.L. (2021). Comparison of 3D scanning versus traditional methods of capturing foot and ankle morphology for the fabrication of orthoses: A systematic review. J. Foot Ankle Res..

[13] Wang J.Z., Wojciechowski E.A., Paine T., Burns J., Cheng T.L. (2025). Feasibility of Designing, Manufacturing and Delivering 3D Printed Ankle-Foot Orthoses: An Updated Systematic Review. J. Foot Ankle Res..

[14] Leith J., Andrysek J., Kuspinar A. (2025). Orthotists’ perspectives on the adjustment of 3D printed ankle foot orthoses: A mixed methods feasibility study. Prosthet. Orthot. Int..

[15] Chen R.K., Jin Y.A., Wensman J., Shih A. (2016). Additive manufacturing of custom orthoses and prostheses-A review. Addit. Manuf..

[16] Raj R., Dixit A.R., Lukaszewski K., Wichniarek R., Rybarczyk J., Kuczko W., Gorski F. (2022). Numerical and Experimental Mechanical Analysis of Additively Manufactured Ankle-Foot Orthoses. Materials.

[17] Dombroski C.E., Balsdon M.E., Froats A. (2014). The use of a low cost 3D scanning and printing tool in the manufacture of custom-made foot orthoses: A preliminary study. BMC Res. Notes.

[18] Silva R., Veloso A., Alves N., Fernandes C., Morouco P. (2022). A Review of Additive Manufacturing Studies for Producing Customized Ankle-Foot Orthoses. Bioengineering.

[19] Atallah H., Qufabz T., Bakhsh H.R., Ferriero G. (2025). The current state of 3D-printed orthoses clinical outcomes: A systematic review. BMC Musculoskelet. Disord..

[20] Farhan M., Wang J.Z., Warncke R., Cheng T.L., Burns J. (2024). Comparison of accuracy and speed between plaster casting, high-cost and low-cost 3D scanners to capture foot, ankle and lower leg morphology of children requiring ankle-foot orthoses. J. Foot Ankle Res..

[21] Silva R., Silva B., Fernandes C., Morouco P., Alves N., Veloso A. (2024). A Review on 3D Scanners Studies for Producing Customized Orthoses. Sensors.

[22] Telfer S., Woodburn J. (2010). The use of 3D surface scanning for the measurement and assessment of the human foot. J. Foot Ankle Res..

[23] Allan J.J., Munteanu S.E., Bonanno D.R., Buldt A.K., Choppin S., Bullas A., Pearce N., Menz H.B. (2023). Methodological and statistical approaches for the assessment of foot shape using three-dimensional foot scanning: A scoping review. J. Foot Ankle Res..

[24] Powers O.A., Palmer J.R., Wilken J.M. (2022). Reliability and validity of 3D limb scanning for ankle-foot orthosis fitting. Prosthet. Orthot. Int..

[25] Totah D., Menon M., Jones-Hershinow C., Barton K., Gates D.H. (2019). The impact of ankle-foot orthosis stiffness on gait: A systematic literature review. Gait Posture.

[26] Kerkum Y.L., Buizer A.I., van den Noort J.C., Becher J.G., Harlaar J., Brehm M.A. (2015). The Effects of Varying Ankle Foot Orthosis Stiffness on Gait in Children with Spastic Cerebral Palsy Who Walk with Excessive Knee Flexion. PLoS ONE.

[27] Vasiliauskaite E., Ielapi A., De Beule M., Van Paepegem W., Deckers J.P., Vermandel M., Forward M., Plasschaert F. (2021). A study on the efficacy of AFO stiffness prescriptions. Disabil. Rehabil. Assist. Technol..

[28] Bielby S.A., Warrick T.J., Benson D., Brooks R.E., Skewes E., Alvarez E., Dunning C., DesJardins J.D. (2010). Trimline severity significantly affects rotational stiffness of ankle-foot orthosis. JPO J. Prosthet. Orthot..

[29] Peng C.X., Tran P., Lalor S., Tirosh D., Rutz E. (2024). Tuning the mechanical responses of 3D-printed ankle-foot orthoses: A numerical study. Int. J. Bioprinting.

[30] Gefen A., Haberman E. (2007). Viscoelastic properties of ovine adipose tissue covering the gluteus muscles. J. Biomech. Eng..

[31] Carroll M., Annabell M.E., Rome K. (2011). Reliability of capturing foot parameters using digital scanning and the neutral suspension casting technique. J. Foot Ankle Res..

