# Supplementary material for: Comparison of Direct 3D Scans and Plaster Casts in Digital Ankle–Foot Orthosis Workflows
Source: Med Sci (Basel). 2026 Sep 19;14(5):589. doi: 10.3390/medsci14050589 (PMC13609648; doi:10.3390/medsci14050589)
Supplement: Supplementary file 1 [file medsci-14-00589-s001.zip › medsci-4528327-supplementary.pdf]

Supplementary Materials

Comparison of Direct 3D Scans and Plaster Casts in Digital Ankle–Foot Orthosis Workflows

Chenxi Peng [1*], Simon Lalor [2], Nasim Shokoohi [2, 3, 4], Ling Chen [1], Erich Rutz [2, 3, 4, 5 and 6*]

1 CSIRO (Commonwealth Scientific and Industrial Research Organisation) Technology, Clayton South, VIC 3169, Australia
2 The Royal Children's Hospital Melbourne, Parkville, Victoria 3052, Australia
3 Department of Paediatrics, The University of Melbourne, Parkville, Victoria 3052, Australia
4 Murdoch Children's Research Institute, Parkville, Victoria 3052, Australia
5 The Hugh Williamson Gait Analysis Laboratory, The Royal Children's Hospital Melbourne, Parkville, Victoria 3052, Australia
6 Medical Faculty, The University of Basel, Basel 4001, Switzerland
* Correspondence: chenxi.peng@csiro.au; erich.rutz@rch.org.au

S1 Ankle proxy definition and sensitivity

S1.1 Qualitative anatomical assessment

To assess the anatomical plausibility of the proportional posterior ankle band, the region mask was visually inspected on all 14 trimmed direct-scan meshes (seven participants, bilateral limbs). For each mesh, height was normalised from 0 to 1 using the minimum and maximum z coordinates of the trimmed scan. The ankle/lower-shank band was defined as 0.12 <= normalised height < 0.32. Within this band, the posterior surface was retained using the 70th percentile of the y coordinate, corresponding to the posterior-most 30% of vertices under the analysis coordinate convention. For visual assessment, the lower 45% of each direct-scan mesh was projected in the mediolateral-height plane (**Figure S1**).

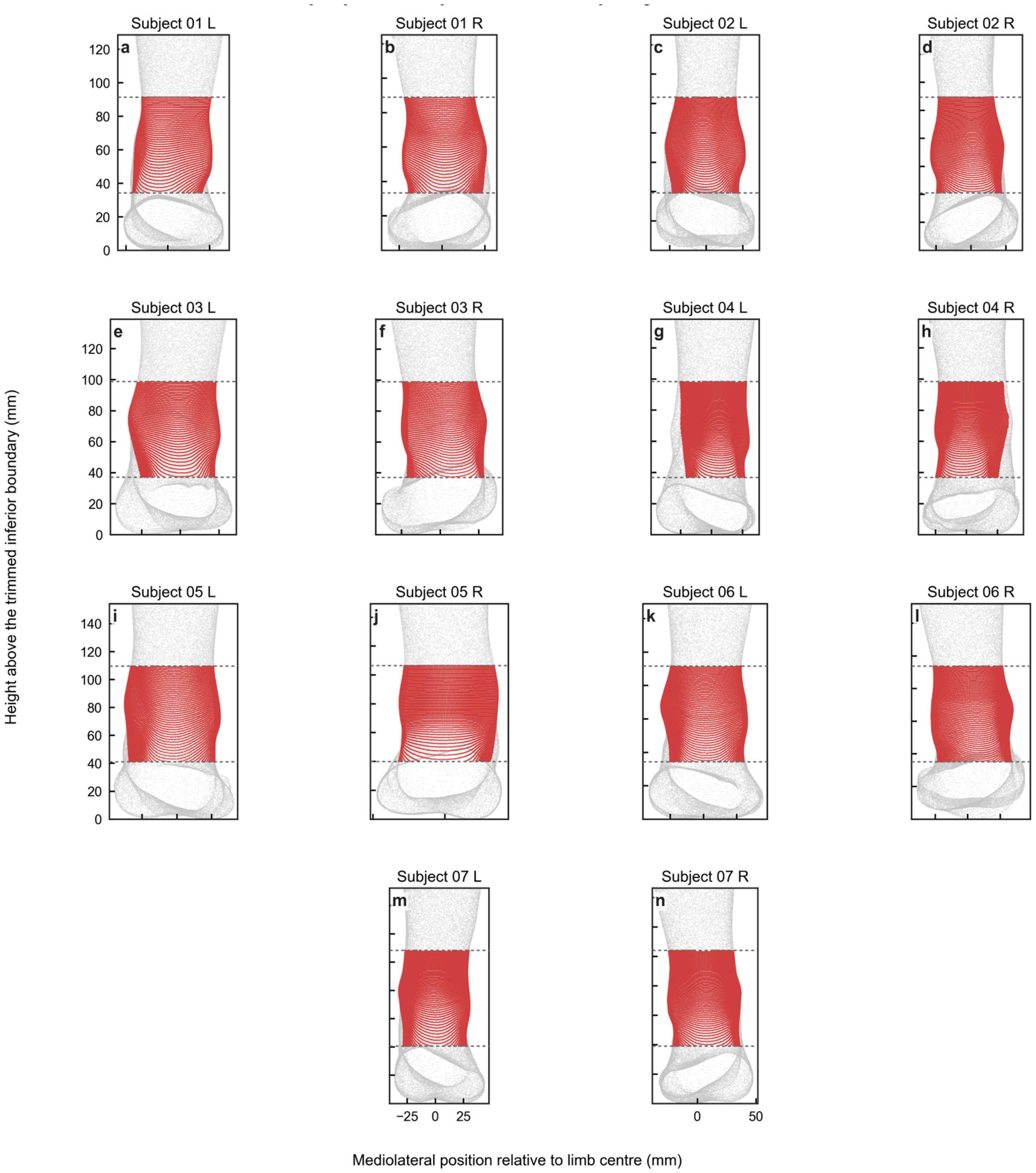

Figure S1. Panels a-n show mediolateral-height projections of the lower 45% of the trimmed direct-scan mesh for the left and right limbs of seven participants. Grey points show the complete trimmed mesh, red points show the posterior ankle proxy, and dashed lines show its inferior and superior proportional boundaries (0.12 and 0.32 of normalised scan height).

S1.2 Sensitivity analysis

Sensitivity to the ankle proxy definition was evaluated across all 14 limbs using all 27 combinations of lower normalised-height boundaries of 0.10, 0.12, and 0.14; upper boundaries of 0.30, 0.32, and 0.34; and posterior retention of 25%, 30%, and 35%. For each limb and scenario, the within-band y-coordinate threshold was recalculated from the trimmed direct-scan vertices, and the corresponding posterior cast vertices were selected. The baseline definition was 0.12–0.32 with the posterior-most 30% retained. Masks were derived from the final whole-limb-registered and trimmed vertex maps. For each limb and definition, the selected cast region underwent centroid matching followed by rigid local ICP registration for up to 25 iterations before cast-to-scan distances were calculated, consistent with the primary regional analysis. Cast–mask overlap with baseline was

quantified using the Jaccard index, defined as the size of the intersection divided by the size of the union.

Figure S2 presents the sensitivity analysis results. After local realignment, the baseline cast-vertex-weighted cohort mean distance was 3.49 mm. One-at-a-time changes in either height boundary or posterior retention altered the cohort mean by no more than 0.11 mm (3.22%). Across all 27 definitions, the largest absolute change in the cohort mean was 0.23 mm (6.55%), the largest absolute change in the weighted mean limb-level P95 was 0.75 mm, and the largest absolute change in the proportion within 2 mm was 6.00 percentage points. Mean limb-level cast–mask Jaccard overlap with baseline ranged from 0.62 to 1.00, and the mean included proportion ranged from 4.34% to 9.65% of trimmed cast vertices.

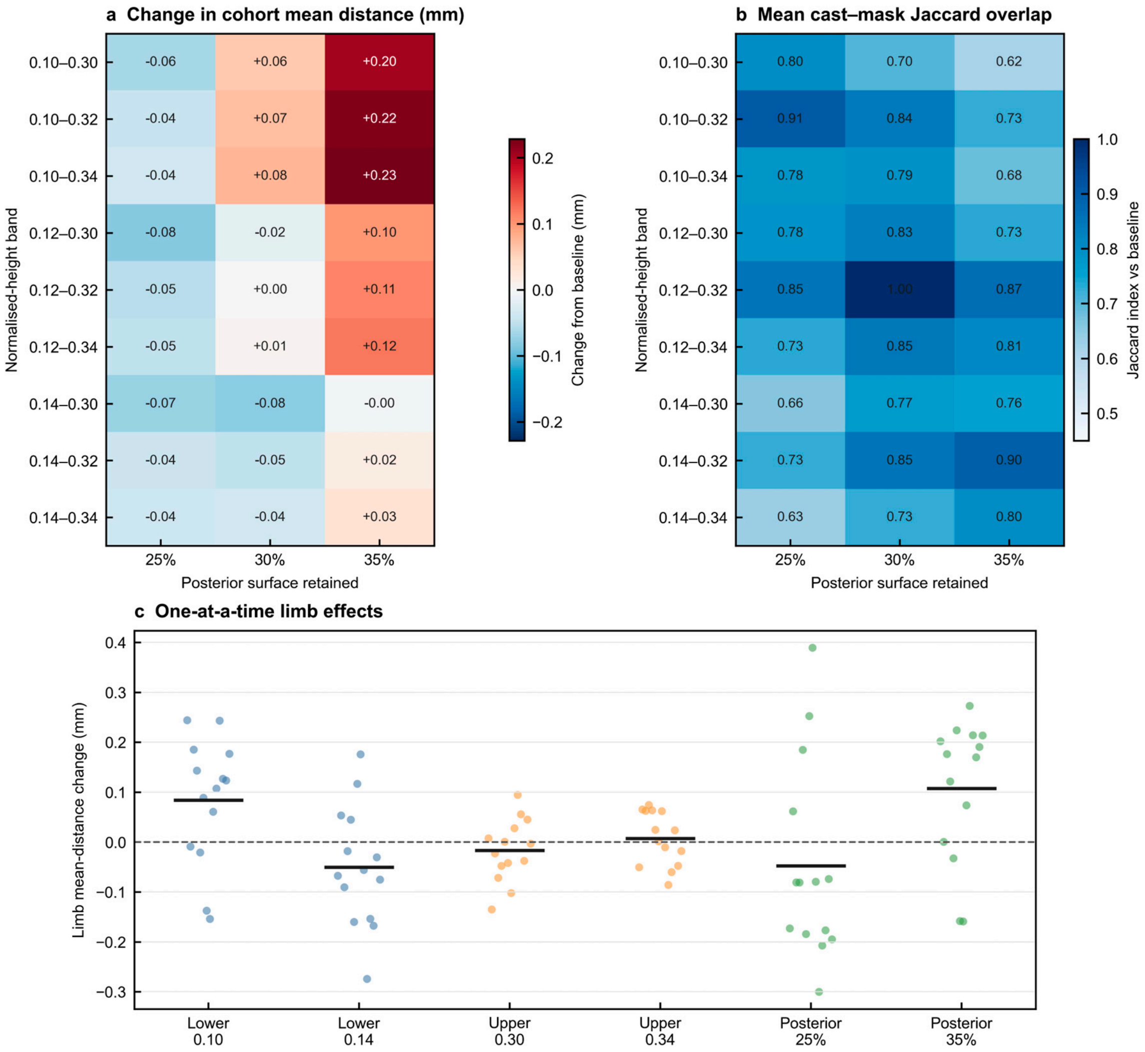

Figure S2. Sensitivity of ankle-region results to the anatomical proxy definition. All 27 combinations of lower normalised-height boundary (0.10, 0.12, or 0.14), upper boundary (0.30, 0.32, or 0.34), and posterior surface retained (25%, 30%, or 35%) were evaluated across 14 limbs. The baseline was 0.12-0.32 with 30% posterior retention. (a) Change in cast-vertex-weighted cohort mean distance relative to baseline. (b) Mean limb-level cast–mask Jaccard overlap with baseline. (c) Limb-level mean-distance changes for one-at-a-time variations; horizontal bars show limb means.

S2 Regional alignment sensitivity

Regional alignment sensitivity was evaluated by comparing distances after whole-limb registration alone with distances after additional independent regional rigid realignment. The analysis included both limbs of seven participants. Final whole-limb-registered and trimmed geometries were analysed using the original foot, ankle, calf and knee definitions and posterior masks. For the whole-limb-only condition, regional distances were obtained without additional centroid translation or ICP. These distances retained regional positional differences together with local surface differences. For the locally realigned condition, each cast region was translated to match the scan centroid and refined using rigid ICP.

Table S1 shows regional mean distance under whole-limb registration and local realignment. The ankle had the highest vertex-weighted mean distance after whole-limb registration only (5.64 mm, bootstrap 95% CI 5.16 to 6.22 mm). The corresponding means were 3.97 mm for the foot, 4.59 mm for the calf and 3.36 mm for the knee. The ankle was the highest-mismatch region in 11 of 14 limbs. Local realignment reduced the mean distance in all comparisons. The mean paired reductions were 1.62 mm for the foot, 2.20 mm for the ankle, 2.10 mm for the calf and 1.06 mm for the knee. The ankle showed the largest absolute reduction, whereas the calf showed the largest proportional reduction. The ankle had the highest cohort mean under both conditions. It ranked highest in 11 of 14 limbs without local realignment and 13 of 14 limbs after local realignment. Thus, regional realignment altered the magnitude of mismatch but preserved the cohort-level ranking. Whole-limb registration and independent regional realignment provide complementary descriptions of geometric disagreement. The former retains relative regional positioning, whereas the latter reduces rigid positional offsets. Residual distances remain dependent on region selection, surface sampling and correspondence.

Table S1. Regional mean distance under whole-limb registration and local realignment (unit in mm).

Region	Whole-limb only mean (95% CI)	Locally realigned mean (95% CI)	Reduction (95% CI)
Foot	3.97 (3.53 to 4.40)	2.34 (2.13 to 2.55)	1.62 (1.30 to 1.98)
Ankle	5.64 (5.16 to 6.22)	3.49 (3.16 to 3.81)	2.20 (1.78 to 2.65)
Calf	4.59 (4.18 to 4.98)	2.48 (2.10 to 2.87)	2.10 (1.80 to 2.38)
Knee	3.36 (2.96 to 3.76)	2.28 (1.92 to 2.60)	1.06 (0.76 to 1.38)

S3. Direction of residual ankle displacement.

Directional analysis assessed whether the cast surface lay inward or outward relative to the direct-scan surface after local rigid ankle realignment. For each cast vertex, c, the nearest point, q, was identified on the direct-scan triangle surface within the corresponding posterior ankle patch. Signed normal displacement, d_n, was calculated as

$$d_n = (c - q) \cdot n(q), \quad (1)$$

where $n(q)$ is the unit outward surface normal at the nearest point. Positive values indicated outward cast displacement relative to the direct scan, whereas negative values indicated inward displacement. Surface normals were recomputed from the mesh geometry after establishing consistent triangle winding. The patch orientation was determined relative to radial vectors extending from the ankle-band centreline. Normals at nearest-surface points were interpolated from vertex normals using barycentric coordinates and normalised to unit length. The corresponding face normal was used when interpolation was degenerate. Displacements within −0.05 to +0.05 mm were classified as near zero. A limb was classified as predominantly outward when at least 60% of its cast vertices had displacement >+0.05 mm. Otherwise, it was classified as predominantly inward. The cohort mean and directional proportions were weighted by the number of cast vertices in each limb.

Table S2 shows results of directional analysis across limbs. Across 143,673 cast vertices, the vertex-weighted mean signed normal displacement was +2.70 mm (bootstrap 95% CI +2.44 to +2.92 mm). Outward displacement occurred at 86.3% of vertices, inward displacement at 13.2%, and near-zero displacement at 0.5%. Limb-level mean displacements ranged from +1.41 to +3.38 mm. All 14 limbs met the predominantly outward criterion. These findings showed a consistent outward tendency across limbs. Nevertheless, the presence of inward vertices demonstrated that residual surface differences were spatially heterogeneous rather than uniformly outward.

Table S2. Signed normal displacement across limbs.

Limb	Mean mm	Outward %	Inward %	Near zero %	Pattern
S01 L	+2.58	85.3	14.2	0.5	outward
S01 R	+1.41	84.3	14.4	1.2	outward
S02 L	+2.21	87.3	12.3	0.4	outward
S02 R	+2.36	86.8	12.8	0.4	outward
S03 L	+2.71	92.4	7.1	0.5	outward
S03 R	+2.87	91.8	7.7	0.5	outward
S04 L	+2.91	83.4	16.0	0.6	outward
S04 R	+3.06	83.6	15.7	0.7	outward
S05 L	+3.00	85.7	14.0	0.3	outward
S05 R	+3.38	80.4	19.3	0.3	outward
S06 L	+2.84	84.2	15.3	0.5	outward
S06 R	+3.16	89.2	10.2	0.6	outward
S07 L	+2.42	86.8	12.7	0.5	outward
S07 R	+2.49	85.3	14.4	0.4	outward
